# Liquid Metal Flexible EMG Gel Electrodes for Gesture Recognition

**DOI:** 10.3390/bios13070692

**Published:** 2023-06-29

**Authors:** Yanru Bai, Xiaoqing Li, Chengcai Zheng, Rui Guo, Xisheng Li

**Affiliations:** 1 School of Automation and Electrical Engineering, University of Science and Technology Beijing, Beijing 100083, China; yrbai@sina.com (Y.B.);; 2 School of Advanced Engineering, University of Science and Technology Beijing, Beijing 100083, China; 3 Department of Biomedical Engineering, Tianjin University, Tianjin 300072, China; 18835258944@163.com

**Keywords:** liquid metal, micro/nanodroplets, EMG electrodes, gesture recognition

## Abstract

Gesture recognition has been playing an increasingly important role in the field of intelligent control and human–computer interaction. Gesture recognition technology based on electromyography (EMG) with high accuracy has been widely applied. However, conventional rigid EMG electrodes do not fit the mechanical properties of human skin. Therefore, rigid EMG electrodes are easily influenced by body movements, and uncomfortable to wear and use for a long time. To solve these problems, a stretchable EMG electrode based on liquid metal nanoparticles was developed in this research. It is conformal with human skin because of its similar mechanical properties to skin. Liquid metal nanoparticles mixed in polymer can be connected to each other to form conductive circuits when pressed by mechanical force. Therefore, this preparation method of liquid metal flexible gel electrodes is low-cost and can be fabricated largely. Moreover, the liquid metal flexible gel electrodes have great stretch ability. Their resistance increases slightly at maximum strain state. Based on these advantages, the flexible gel electrodes are applied to arm to collect EMG signals generated by human hand movements. In addition, the signals are analyzed by artificial intelligence algorithm to realize accurate gesture recognition.

## 1. Introduction

Flexible electrodes are a kind of key flexible electronic device that can be used in human–machine interface, biomedical monitoring, and energy storage [1,2,3,4]. They are usually composed of flexible substrates and flexible conductive layers, so they can adapt to various complex surfaces. However, the performance of flexible electrodes still faces some challenges, such as the difficulty in balancing the stretchability and conductivity of conductive materials, as well as the durability of the electrode and substrate interface [5,6,7]. Therefore, developing better flexible electrode materials and structures is of great significance for achieving high-performance flexible electronic devices.

Multiple strategies have been proposed for the flexibility in the conductive part of flexible electrodes. For example, nanoconductive materials such as carbon nanotubes, graphene, and silver nanoparticles can be used as conductive layers. Their high flexibility can meet the requirements of flexible electrodes, but their conductivity is often low [8,9,10]. Moreover, structural engineered rigid metal films with geometric shapes such as snakes, buckles, or rings can achieve additional scalability through local deformation. However, the tensile strength of rigid metal materials is low, making it difficult to adapt to significant stretching deformation [11,12,13]. In addition, organic conductive materials, such as poly (3,4-ethylenedioxythiophene) (PEDOT) and Polyaniline (PANI), or other conductive polymer materials can be used as the conductive layer of flexible electrodes. However, the organic conductive polymer materials usually have low chemical stability, which is prone to oxidation, reduction, hydrolysis, and other reactions, leading to the reduction in conductivity [14,15].

In recent years, room temperature liquid metal (LM) based on gallium has been proven to be an ideal conductor for soft electronics due to its metal conductivity, infinite deformation, and low toxicity [16,17,18]. There are currently multiple strategies available for preparing LM flexible electrodes. For example, studies have shown that various soft electronic devices can be designed by injecting LM into elastomers such as polydimethylsiloxane (PDMS) [19,20,21]. There are also studies on designing multi-functional soft electronic equipment by patterning LM on hydrogel or silica gel as conductive interconnection [22,23]. The above methods can achieve high-precision manufacturing of LM circuits by manufacturing microchannels or templates with microstructures. However, the manufacturing process of these microchannels and templates is complex, costly, and requires expensive equipment. In addition, the above methods can only be used to manufacture circuits of specific shapes and sizes, making it difficult to achieve large-scale and customized flexible circuits.

Recently, research has proposed a strategy of mixing LM with polymers to create LM complex, which can effectively reduce the surface tension of LM and achieve circuit patterning on various substrates [24,25]. LM droplets can form conductive pathways through mechanical force destruction, breaking away from the limitations of high-precision microchannels or templates. Here, we propose a simple and convenient manufacturing method to create LM flexible gel electrodes, and the flexible electrodes have high stretchability, with a maximum stretchability of up to 100%. We mixed LM in stretchable PDMS to form LM complex. Afterwards, the barrier structure between LM droplets was destroyed by mechanical force, forming a conductive pathway. This method only requires adjusting the movement of the rigid needle to directly achieve patterning of LM circuits on PDMS, with the traversing precision of up to 100 μm. We have utilized these LM flexible gel electrodes to achieve the collection of human EMG signals, and the flexible gel electrodes can be conformally applied to the human skin, improving the stability of signal collection and wearing comfort level. Hydrogels are electrically conductive and flexible, providing tight adhesion to the skin. Various hydrogel materials with adhesion mechanisms have been developed [26,27,28,29,30]. Here, conductive hydrogels are covered with LM conductor surfaces and used to provide a strong adhesion to the skin. A non-Newtonian fluid (polyborosiloxane, PBS) is overlaid on the surface of PDMS to enhance the adhesion of PDMS to the skin. Finally, we achieved high accuracy recognition of gestures by constructing a surface EMG signal processing algorithm based on machine learning.

## 2. Results and Discussions

**Figure 1:**
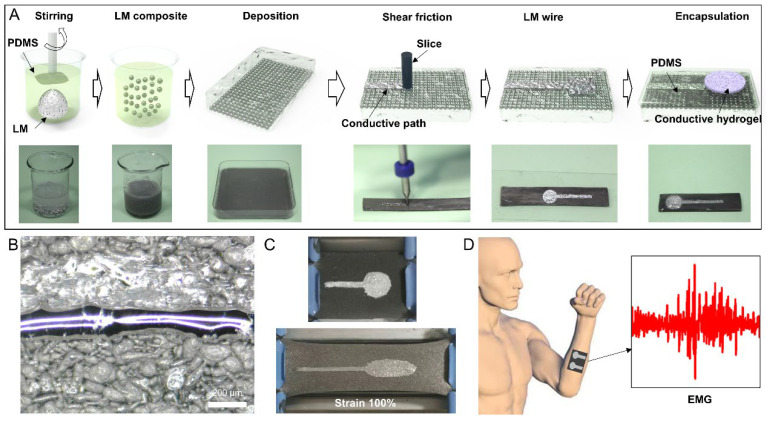
Preparation method, demonstration, and application of the LM EMG gel electrode. (**A**) Preparation method for the LM EMG gel electrode. (**B**) LM wire with the smallest linewidth. (**C**) Stretchability demonstration of a flexible electrode. (**D**) Flexible EMG gel electrode applied to human body surface EMG detection.

**Figure 2:**
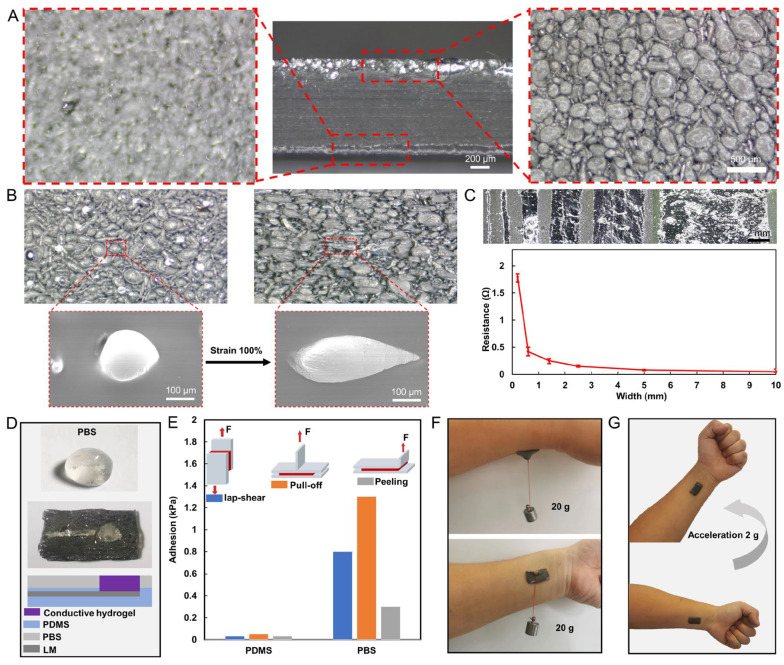
Microscopic characterization and adhesion properties of the LM EMG gel electrode. (**A**) Optical microscopic images of the bottom and top layers of LMC films. (**B**) Optical and electron microscopic images of LMC films before and after stretching. (**C**) LM wires of different widths and their resistances. (**D**) Structure of PBS-enhanced high-adhesion electrode patch. (**E**) Adhesion of PDMS with and without PBS film modification to skin under three peel tests (lap-shear, peeling, and pull-off tests). (**F**) The patch suspended tangentially with 20 g weight and 20 g weight suspended in the normal direction. (**G**) The patch stayed firmly attached to the skin under 2 g acceleration.

In this study, a two-step preparation method was proposed for enhancing the stretchability and minimizing the thickness of EMG electrodes. The first step involves the breaking of LM into micron liquid droplets, which are then encapsulated in stretchable polydimethylsiloxane (PDMS). Subsequently, the dispersed LM droplets are electrically connected using a simple mechanical sintering method. The preparation process of the LM circuit is illustrated in Figure 1A, and detailed steps are presented in Section 3. The preparation process commenced with the preparation of LM complex (LMC), which was fully mixed with PDMS to form the complex coated with PDMS on the surface of LM droplets. The PDMS layer rendered the LMC film electrically insulated. Next, the LMC was poured into a mold, where the density of the LM caused it to accumulate at the bottom of the polymer due to gravity. Subsequently, a metal needle tip was employed to squeeze the bottom of the LMC film, break the oxide layer on the LM surface, and connect the droplets to transform the LMC film into a LM circuit, as depicted in Figure 2B. An LM wire with the smallest linewidth of 100 μm was realized by moving a single rigid needle. Using this method, an EMG electrode was prepared, which exhibited good stretchability, with the maximum strain rate reaching 100%, as demonstrated in Figure 1C. To transmit EMG signals, conductive hydrogels were applied to the electrode points. Here, the hydrogels were covered on the LM surface instead of PDMS. Previous study has shown that the hydrogel forms hydrogen bonds with the oxide film on the LM surface, thus achieving a high adhesion between them [22]. Furthermore, to protect the extruded LM wire from damage, the electrode was also covered with a second PDMS film layer, which was generated by casting PDMS prepolymer and curing. The entire LMC film measured only 1 mm. The PDMS demonstrated excellent compliance and therefore adapted to the curved surface of the skin. Additionally, almost no LM droplets exist on the upper layer of the LMC film, ensuring that the LM does not leak out from the upper surface of the electrode array. The structure thus possesses excellent stability and can meet long-term use requirements.

A wide range of LM wires with different wire widths can be manufactured using LMC films for various integrated levels in electronic device manufacturing. The stratification of LM droplets in a thin film is depicted in Figure 2A. The LM droplets accumulate at the bottom of the film, forming a 150-μm-thick layer of droplets, while the upper layer is the PDMS layer with 850 μm thickness, which provides effective protection against leakage of LM droplets from the upper layer. SEM images from the bottom of the LMC film demonstrate that more than 90% of the particle mass is between 45 and 400 µm in diameter. The stretchability of PDMS films is not limited by the fluidity of LM droplets. As shown in Figure 2B, the diameter of LM droplets was increased from 211 μm in the original length to 442 μm in the 100% strain ratio state. The LM leaked out of the PDMS and fused with the surrounding LM to form a conductive path on the track squeezed by the rigid needle. The linewidth of this conductive path was adjusted by controlling the trajectory of the rigid needle. LM wires with wire widths ranging from 200 μm to 1 cm were prepared. The resistance of LM wires with different widths was measured, as shown in Figure 2C. It is observed that as the linewidth increases, the resistance value decreases. The LM wire with the smallest linewidth resistance value is 1.8 Ω, while the widest wire resistance value is 0.048 Ω. The resistance values are much larger than the linewidth ratio. This is due to the non-uniform cross section shape of the wires with different linewidths, which results in local abnormal resistance values.

The situation was found that PDMS had poor adhesion to the hydrogel and the hydrogels tended to peel off from the electrode patch. In addition, the limited adhesion of PDMS to the skin resulted in easy peeling of the electrode patch from the skin during limb movement. To solve these problems, PBS was made into a film with a thickness of 200 μm and was applied to the PDMS surface. The PBS film has high adhesion to both PDMS and hydrogel, which can improve the stability of the electrode structure, as shown in Figure 2D. We evaluated the adhesion of the PBS-enhanced high-adhesion electrode patch by carrying out three different types of force experiments to measure (1) the shear strength by lap-shear tests; (2) interfacial toughness by peel tests; and (3) tensile strength by pull-off tests. was Two PDMS encapsulated electrode patches (1 cm × 2 cm, with/without PBS film) were applied to the skin separately. An extensometer was clamped to the position of the electrode patch as shown in Figure 2E and lifted upward at a speed of 50 mm/min until the electrode patch was detached from the skin. Throughout the process, the tensile force on the stretching table was recorded, the maximum of which was considered as the adhesion. The results in Figure 2E show that the adhesion of the electrode patch covered with PBS film (0.8, 1.3, and 0.3 kPa) to the skin is significantly higher than that of the electrode patch not covered with PBS film (0.03, 0.05, and 0.03 kPa). Moreover, as illustrated in Figure 2F, the PBS-enhanced high-adhesion electrode patch was verified experimentally by suspending 20 g tangentially and 20 g in the normal direction, and it could be stably hanged with skin. Finally, the patch stayed firmly attached to the skin under 2 g acceleration.

**Figure 3:**
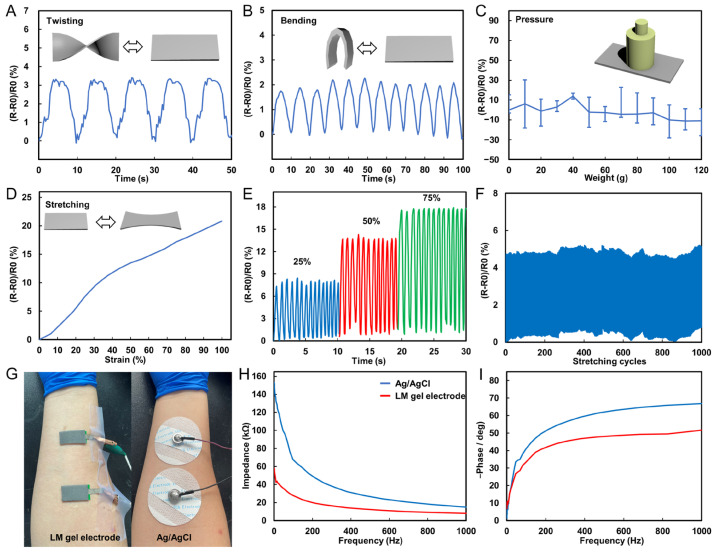
Electrical properties of LM flexible conductors. (**A**) Resistance change curve of LM wire in the process of twist for 5 cycles. (**B**) Resistance change curve of LM wire in the process of bending 10 cycles. (**C**) Resistance change curve of LM wire under different pressures. (**D**) Resistance curves of LM wires under different strain states. (**E**) Resistance change curve of LM wire in the process of being stretched 10 times under different strain states. (**F**) Resistance change curve of LM wire in the process of stretching 1000 cycles. (**G**) Picture of LM EMG gel electrodes and commercial Ag/AgCl electrodes applied in sequence at the same position on the arm. (**H**) Impedance of LM EMG gel electrodes and commercial Ag/AgCl electrodes at different frequencies. (**I**) Phase response of LM EMG gel electrodes and commercial Ag/AgCl electrodes at different frequencies.

In daily life, human skin undergoes many twists, bends, and stretches, and thus the electrical properties of LM flexible wires under various deformation conditions need to be tested to assess whether they can meet the requirements of long-term wearing applications. To this end, some LM wires (width of 1 mm, length of 5 cm) were prepared and their electrical properties were observed by torsional and bending experiments. Specifically, the wire was twisted 180° for 5 cycles and bent 180° for 10 cycles at 0.1 Hz frequency, and the resistance of the wire during the bending and twisting process was recorded. The resistance in the twisting process is shown in Figure 3A, where it can be observed that the twisting value of the wire leads to a slight increase in the resistance and maintains a stable change rule in multiple twisting cycles, with the maximum resistance change rate being only 3.4%. Similarly, bending would also lead to a slight increase in resistance and maintain a stable change rule, with the maximum resistance change rate being only 2.1%, as shown in Figure 3B. The experimental results show that the resistance of the wire undergoes a slight change during the twisting and bending cycles. Due to the impedance of the EMG electrode interface, which can reach several tens of kiloohms, the slight resistance variation in the EMG electrode wires will not affect the electrical performance of the EMG electrodes. This ensures the electrical stability of the electrodes in deformed states.

Moreover, different weights (0–120 g) were placed on the LM wire, and the resistance under different pressures was recorded, as shown in Figure 3C. Despite resistance changes of up to 14%, its maximum resistance change is only 0.46 Ω. The results show that the wire resistance is stable under different pressures. In addition, strain experiments were conducted on the wires by stretching them to different conditions and recording their corresponding resistance. It is found that with the increase in the strain rate, the resistance increases significantly, and the resistance change rate under the maximum strain state (100%) can reach 21%, as shown in Figure 3D. The wire was stretched to different conditions (25, 50, and 75%) and repeated for 10 cycles. The experimental results show that the wire can maintain a stable resistance change rate in each cycle, indicating that the electrical performance of the wire has good stability for strain, as shown in Figure 3E. Finally, a long-term stability test for the tensile experiment was conducted by repeatedly stretching the wire 1000 times at 20% stretch rate, and recording the resistance of the wire during the stretching process. The results in Figure 3F show that the resistance of the wire did not change significantly after multiple stretches, indicating that the electrical performance of the wire has good stability in long-term use. The above experiments demonstrate that the stretchable wire can transmit electrical signals stably in the case of frequent deformation and has a good application prospect. Additionally, this experiment proves the long-term stability of the stretchable wire, even in long-term use, its resistance change is not obvious, so it can be applied to some long-term use occasions.

The monitoring of muscle activity through real-time recording of muscle potential signals with flexible EMG electrodes has become a significant technique. Prior to using flexible EMG electrodes, their electrical impedance should be tested. To assess the skin contact impedance of EMG electrodes, we attached the electrodes to a signal generator and applied them to the same locations on the upper limb of a volunteer as commercial Ag/AgCl electrodes (Figure 3G). The impedance of the electrodes was tested at different frequencies ranging from 1 Hz to 1000 Hz. The experimental results demonstrate that the flexible EMG electrodes have a lower electrical impedance than commercial Ag/AgCl electrodes in the range of myoelectric frequencies (Figure 3H). Additionally, we conducted tests on the phase response of the electrodes. The experimental results in Figure 3I show that the phase response of the electrodes is more stable than that of commercial electrodes. Based on the test results, we believe that the flexible EMG electrodes have excellent electrical performance. Their signal impedance and phase response are both outstanding, making them highly useful in the monitoring and evaluation of human muscle movement.

**Figure 4:**
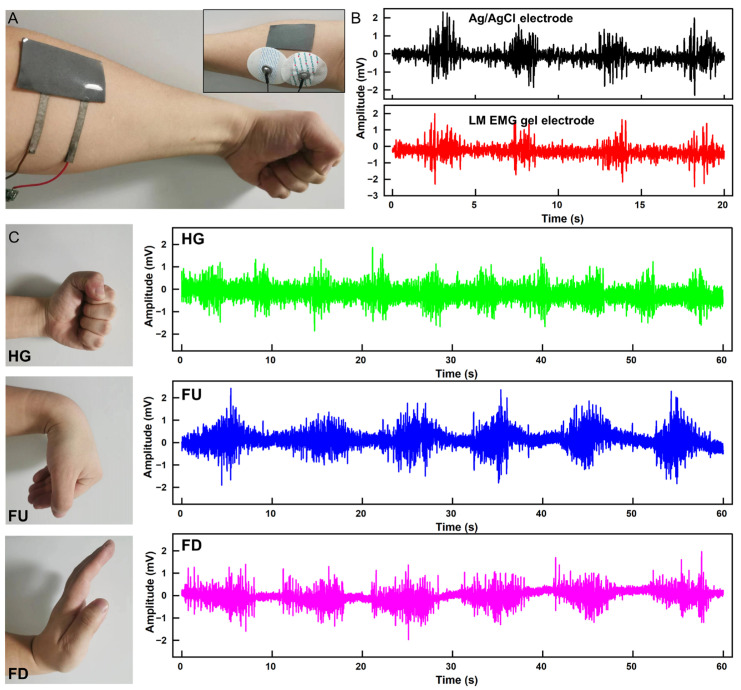
Acquisition of EMG signal. (**A**) LM EMG gel electrodes and commercial electrodes applied to the skin simultaneously. (**B**) EMG signals collected by LM EMG gel electrodes (red) and commercial EMG electrodes (black). (**C**) EMG signals of three gestures collected by LM EMG gel electrodes.

We fabricated two separate electrodes on a liquid metal complex film (6 cm × 3.5 cm) to allow for easy mounting of the electrodes and a consistent distance between the two electrodes, as show in Figure 4A. The performance of the flexible EMG electrode was compared with commercial Ag/AgCl electrode patches in the acquisition of EMG signals. The results in Figure 4B demonstrate that the two electrodes perform similarly in the acquisition of EMG signals. Furthermore, the flexible EMG electrodes were placed on the forearms of a healthy adult, and EMG signals were collected for three gestures (Hand Grasp, HG; Finger Up, FU; Finger Down, FD), each for 1 min. Following the collection, the signals were processed to filter out baseline drift and power frequency interference, as shown in Figure 4C.

**Figure 5:**
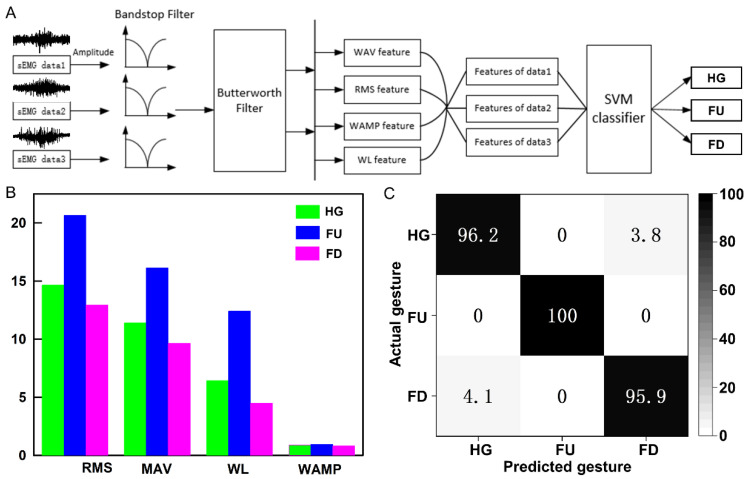
Gesture recognition based on machine learning. (**A**) Processing workflow for EMG signals in gesture recognition. (**B**) Four feature values for the EMG signal activity segments corresponding to three gestures. (**C**) Classification confusion matrix for three gestures.

Finally, we implemented gesture recognition using a machine learning algorithm based on EMG signals. The process of gesture recognition includes EMG signal acquisition and preprocessing, extraction of the onset position of EMG signal activity segments, extraction of features from EMG signal activity segments, and the establishment of a gesture recognition classification model, as shown in Figure 5A. First, we collected single-channel EMG data for three types of gestures (HG, FU, and FD). Prior to the experiment, EMG sensors were worn on the volunteers’ right arm. During the experiment, volunteers sat in a comfortable chair and performed the three gestures with their right hand. Each gesture lasted for 10 s, repeated 50 times, resulting in a total of 150 samples. Initially, we preprocessed the EMG data to remove baseline drift, motion artifacts, and power line interference. Since EMG signals exhibit larger amplitudes during motion execution and smaller amplitudes during rest, we extracted the start and end points of EMG signal activity segments by comparing the energy information of the signals. The key to implementing gesture recognition based on EMG lies in constructing feature extraction and classifier for EMG. In this study, we extracted four feature values for different gesture EMG signal activity segments: root mean square (RMS), mean absolute value (MAV), waveform length (WL), and Wilson amplitude (WAMP), as shown in Figure 5B. It is evident that there are significant differences in feature values among different gesture actions.

Finally, we employed a support vector machine (SVM) for model training. A total of 70% of the feature vectors were randomly selected for training, while the remaining 30% were used for testing. The classification results are presented in the form of a confusion matrix in Figure 5C. These results demonstrate that the SVM classifier achieves high accuracy in recognizing the three gestures. The recognition rate for the FU gesture reaches up to 100%. The recognition rates for the HG and FD gestures are slightly lower at 96.2% and 95.9%, respectively. The average recognition rate for the three gestures is above 97%. Therefore, this classification algorithm is effective and can accurately determine the gesture action based on the input EMG signals.

## 3. Materials and Methods

**Preparation of liquid metal composite.** Gallium and indium (99.99%, Anhui Minor New Materials Co., Ltd., Chuzhou, China) were mixed together in the ratio of 75.5: 24.5 by weight. Then, they were heated at 200 °C for 2 h to obtain liquid metal (EGaIn). Later, 90 g PDMS (Sylgard 184, Dow Corning, Midland, MI, USA) and 10 g EGaIn were added into a glass beaker, and stirred with a glass rod at a stirring speed of 30–50 rpm for 20 min to obtain stabilized liquid metal composite. Then, the liquid metal composite was poured onto a plexiglass substrate at room temperature. After being left to stand for 5 h, the majority of the liquid metal microdroplets had settled at the bottom of the mixture. Subsequently, the liquid metal composite was placed in a constant temperature heating oven (80 °C, 30 min) to allow for the curing of the PDMS, resulting in the formation of a layered liquid metal composite film. This description is presented in the style of academic research writing.

**Preparation of liquid metal electrode.** Firstly, a rigid fine needle was fixed onto a two-dimensional moving platform. Next, the bottom layer of the liquid metal composite film was positioned facing upwards below the needle. Subsequently, the needle was moved along a predetermined path over the film. The liquid metal microdroplets were ruptured under the pressure of the needle and fused with adjacent droplets, thus forming a conductive pathway. Using this method, an electrode was produced. A circular conductive hydrogel patch (Polyacrylic acid based conductive gel, Weaver and Company, Houston, TX, USA) was then affixed onto the electrode point. Finally, a PDMS precursor was poured onto the surface of the electrode array to achieve encapsulation. The entire fabrication process of the electrode array is depicted in Figure 1B. The PBS was prepared by mixing hydroxylterminated PDMS (PDMS-OH) precursor (18,000–22,000 cSt, 5 g) and boric acid (2.42 mg) according to the stoichiometric ratio (1:1). After that, the mixture was stirred at room temperature for 2 h and then heated in vacuum at 120 °C for 12 h.

**Structural characterization.** The surface morphologies of liquid metal composite before and after mechanical damage were obtained by scanning electron microscopy (SEM, Apreo, Thermo Fisher Technology Co., Ltd., Waltham, MA, USA). The mechanical properties of Two PDMS encapsulated electrode patches (1 cm × 2 cm, with/without PBS film) were measured by a high-speed extensometer (KJ-1065A; strain speed, 0.01–500 mm/min; detection precision, 0.01 to 500 N).

**Electronic Measurement.** The resistances of liquid metal lines (length: 5 cm, width: 0.1/0.2/0.4 mm) were measured using a digital multimeter (Keithley 2002, Tektronix Inc., Beaverton, OR, USA). To avoid the influence of contact resistance, a four-terminal method was employed. The electrical properties of the samples (length: 5 cm, with: 1.5 mm) under different stretching states were investigated by a stepper motor (J-5718HB4, Yisheng, Inc., Hangzhou, China) with clamping device.

**EMG collecting system.** An analog front-end amplifier (ADS1298, Texas Instruments, Inc., Dallas, TX, USA) was used to collect EMG signals. A Bluetooth system-on-chip (nRF52832, Nordic Semiconductor Co., Ltd., Trondheim, Norway) was used for signal processing and transmission.

## 4. Conclusions

The attention of researchers has increasingly turned to the use of flexible EMG electrodes as a new type of EMG collector due to their simple manufacturing process, low cost, and flexibility. In this study, it was demonstrated that the LM based flexible EMG electrodes had comparable performance to commercial Ag/AgCl electrode patches in the collection of EMG signals. The use of flexible EMG electrodes as a substitute for traditional commercial electrodes for capturing muscle activity during gestures was found to be effective. This is particularly crucial for applications that require long-term monitoring of muscle activity, given the greater comfort and reduced invasiveness of these electrodes. Additionally, this research demonstrated the promising application prospects of combining comfortable wearable flexible electrodes with machine learning techniques for the recognition of various gestures, offering broad potential in the field of human–computer interaction.

However, it is important to note that this study only investigated three gestures, and further research is required to determine the suitability of this type of electrodes for other gestures or activities. Subsequent research must examine the potential of LM based flexible EMG electrodes in practical applications, focusing on their effectiveness over an extended period, as well as in more dynamic and complex motions compared to traditional commercial electrodes. Additionally, it is critical to examine the effect of electrode placement and clothing on performance. In conclusion, this study suggests that the LM based flexible EMG electrodes could serve as a promising alternative to traditional commercial electrodes and require further investigation to explore their potential for various applications.

## Data Availability

Not applicable.
